# Right-sided Bochdalek hernia containing retroperitoneal fat in the elderly: report of a case

**DOI:** 10.1186/s40792-019-0637-2

**Published:** 2019-05-17

**Authors:** Michihito Toda, Aya Yamamoto, Takashi Iwata

**Affiliations:** Departments of General Thoracic Surgery, Kansai Rosai Hospital, Japan Organization of Occupational Health and Safety, Inabaso 3-1-69, Amagasaki, Hyogo 660-8511 Japan

**Keywords:** Bochdalek hernia, Adult diaphragmatic hernia, Surgery

## Abstract

**Background:**

Most cases of Bochdalek hernias are diagnosed during the neonatal period and arise on the left side. We report a rare case of a right-sided Bochdalek hernia in an elderly patient.

**Case presentation:**

A 72-year-old man presented with chest tightness and nausea. He had no history of thoracic and abdominal trauma. Preoperative CT scan showed a well-circumscribed mass in the right thoracic cavity of 28-cm diameter compressing the right lower lobe. The mass was mostly fat component and seemed to connect with retroperitoneal fat. We made some diagnoses: lipoma, liposarcoma, and diaphragmatic hernia. Surgical resection was performed by thoracotomy so as to resect the mass and repair the defect of the diaphragm. The mass seemed to be retroperitoneal fat which escaped from the hernia orifice. The neck of the mass was separated by a vessel-sealing device immediately above the hernia orifice. The defect of the diaphragm was repaired by direct suturing after completion of resection. Microscopic pathologic examination showed that the mass was maturated fat tissue. Four months postoperatively, there was no evidence of recurrence of the hernia.

**Conclusions:**

The diagnosis of an adult Bochdalek hernia is often difficult, so it is important to consider the examination carefully and to determine the better surgical procedure.

## Background

Bochdalek hernias which are most common types of diaphragmatic hernia comprise 90% of congenital diaphragmatic hernias [1]. Because most cases of Bochdalek hernias are diagnosed during the neonatal period, diagnosis in adults is rare [2]. An adult Bochdalek hernia (ABH) is usually caused by a state of increased intra-abdominal pressure, such as pregnancy and operations under the pneumoperitoneum [3]. Around 80–90% of Bochdalek hernias arise on the left side [4]. There are currently fewer than 100 cases of Bochdalek hernias reported in adults in the literature, and only about 20 cases involving right-sided hernias [5, 6].

We here present a rare case of a right-sided Bochdalek hernia in an elderly patient that was resected via a posterior lateral incision and discuss the clinical presentation and management of ABH.

## Case presentation

**Fig. 1:**
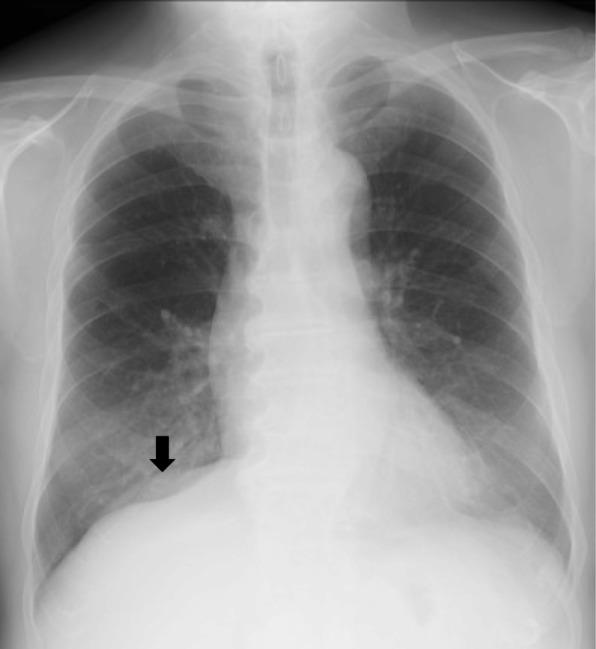
A chest X-ray film showing a double line on the right diaphragm (arrow)

**Fig. 2:**
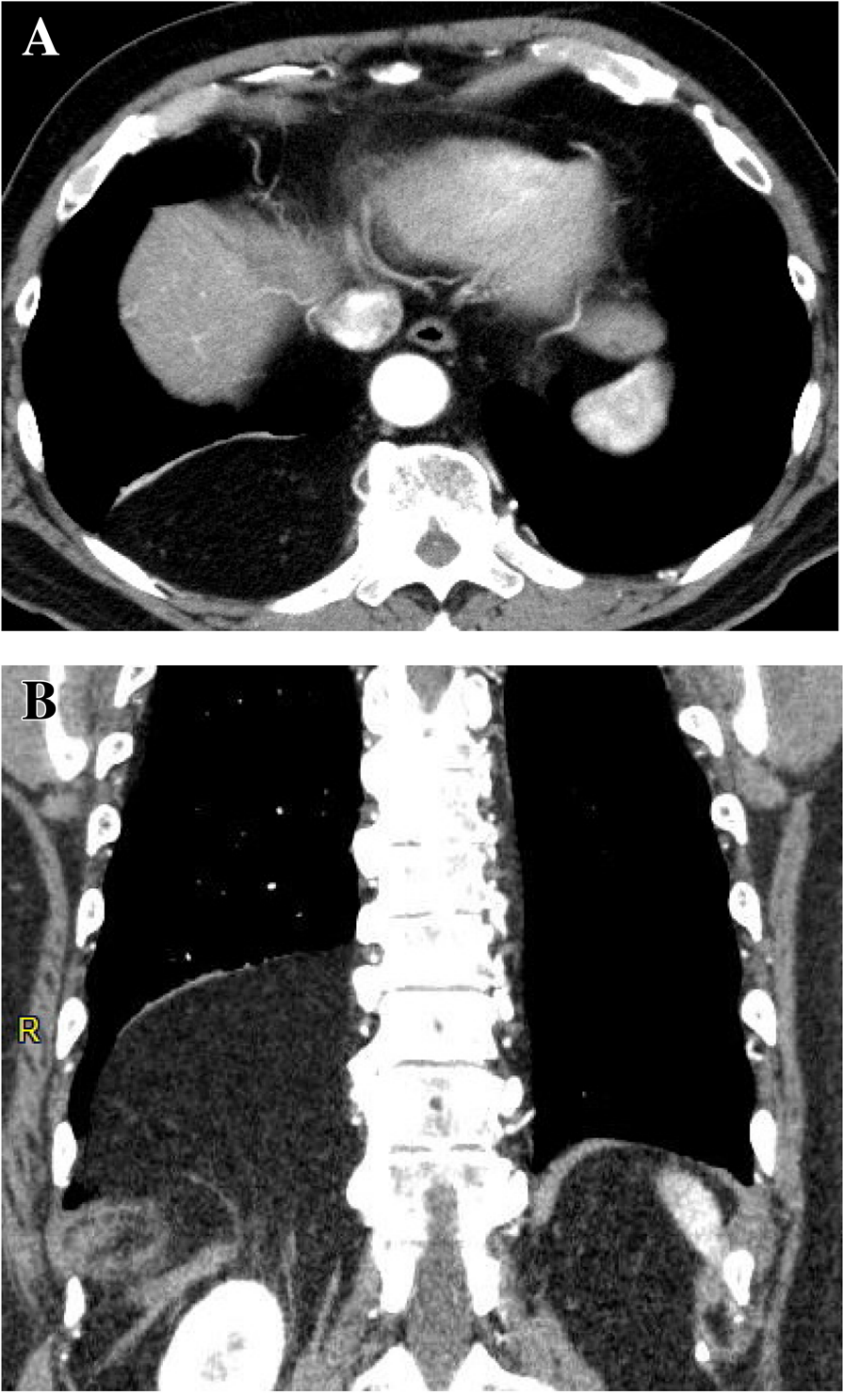
Chest CT images showing **a** a well-circumscribed mass in the right dorsal thoracic space measuring 28 × 9× 10 cm and compressing the right lower lobe. **b** The tumor is mostly composed of fatty density and seemed to connect with retroperitoneal fat

**Fig. 3:**
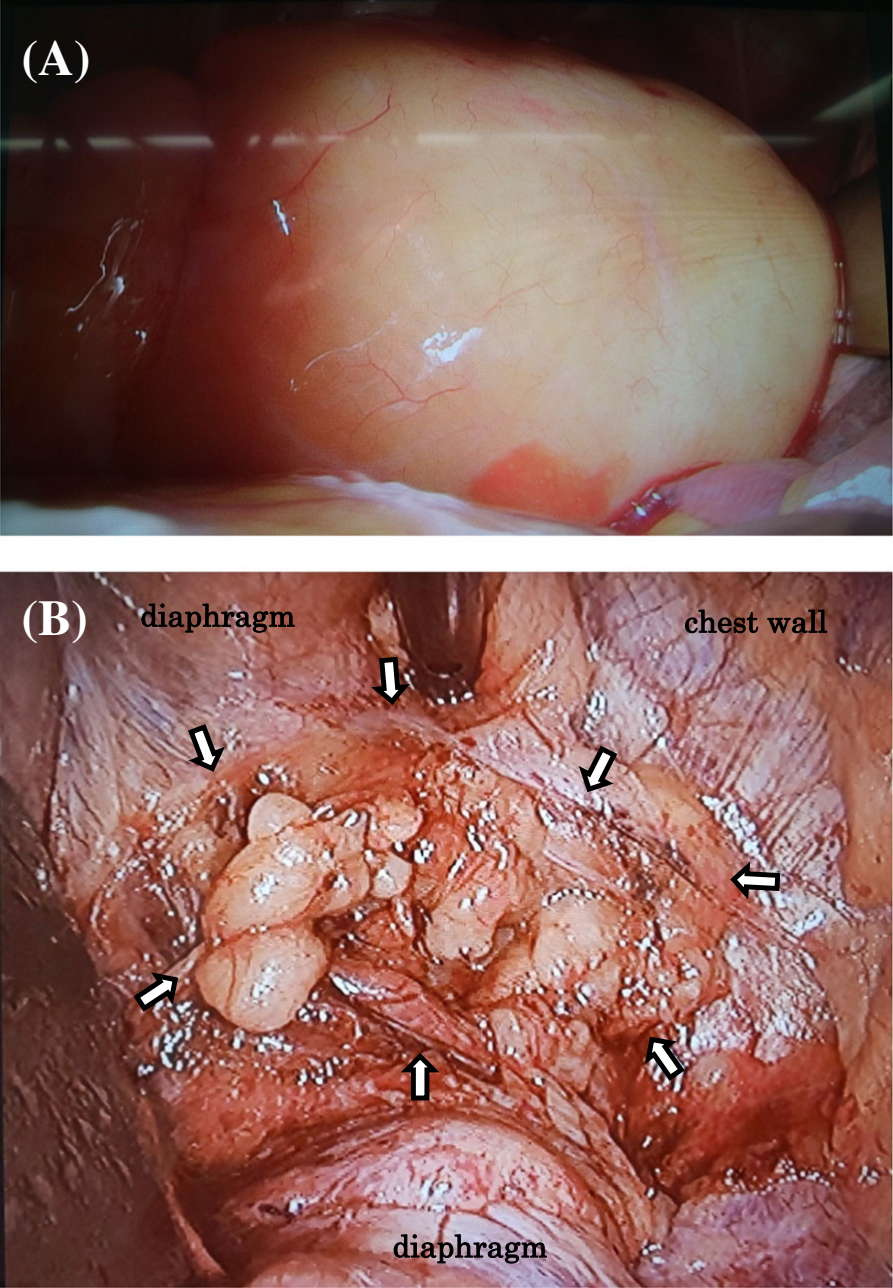
**a** A fatty mass of 28 cm in size, which seems to be retroperitoneal fat, slid into the thoracic cavity. **b** The hernia orifice (surrounded by white arrows) is composed of the right lumbocostal triangle

**Fig. 4:**
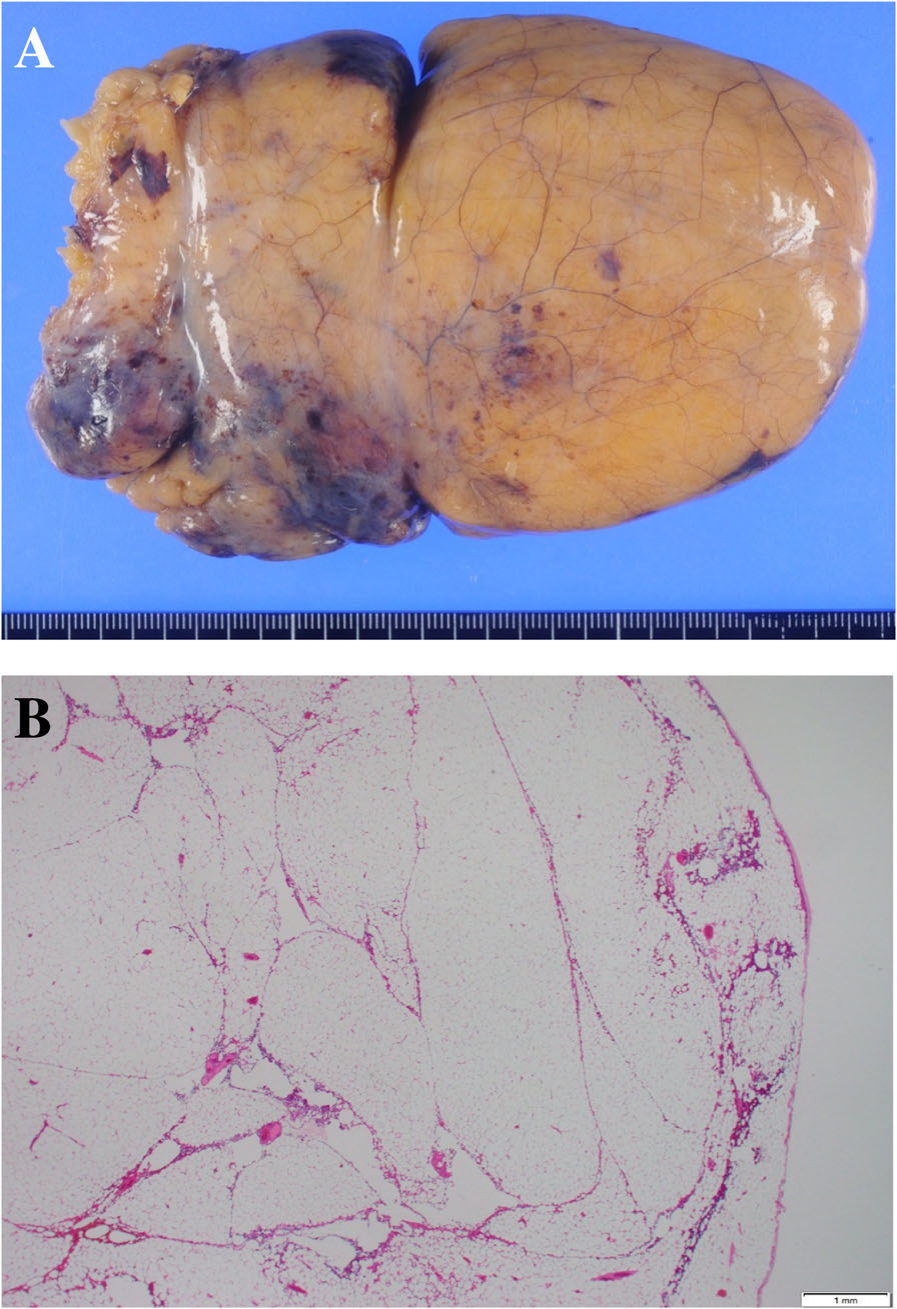
**a** The tumor measured 28 cm × 9.7 cm × 9.5 cm. **b** Histological examination revealed maturated fat tissue

A 72-year-old slightly obese man with a body mass index of 28.4 presented to our hospital for chest tightness and nausea for 2 weeks. There was no history of thoracic and abdominal trauma. A chest X-ray film revealed a double line on the right diaphragm (Fig. 1). Chest CT demonstrated a well-circumscribed mass in the right thoracic cavity measuring 28 cm × 9 cm × 10 cm that was compressing the right lower lobe (Fig. 2a). The mass comprised mostly fatty tissue, and any other organs such as intestinal tract were not included in the mass (Fig. 2b). The results of blood chemistry studies, including tumor markers, were within normal ranges. Thus, the following differential diagnosis were considered: lipoma, liposarcoma, and diaphragmatic hernia. Surgery was performed for diagnosis and treatment via a small lateral thoracotomy via the seventh intercostal space with thoracoscopic assistance. A retroperitoneal fat pad of 28 cm in size was slid into the thoracic cavity from the right lumbocostal triangle, as the hernia orifice (Fig. 3a, b). The size of orifice was about 8 × 5 cm. We transected the neck of the fat pad above the orifice, because the hernia content in the thoracic cavity was larger than the orifice and difficult to reduce. Several feeding arteries contained in the stem were dissected by a vessel-sealing device. The orifice was closed by suturing the surrounding diaphragmatic muscle and the chest wall. The collapsed lung could be re-expanded by positive pressure ventilation without developing acute lung edema. The operation time was 112 min and the total blood loss 220 g. The fat pad measured 28 × 9.7 × 9.5 cm (Fig. 4a). Histological examination revealed maturated fat tissue (Fig. 4b). The chest drain was removed on the first postoperative day. Postoperative chest X-ray films demonstrated progressive re-expansion of the right lower lobes, which had been collapsed preoperatively. The patient was discharged on the third postoperative day. Not only his dyspnea on effort but also his stiff neck improved considerably. When last seen at his 4-month follow-up visit, he reported a good quality of life and there was no evidence of recurrence.

## Discussion

Diagnosis of ABH is not easy. A misdiagnosis rate has been reported as 38% by Thomas and Kapur [7]. ABH onset is considered to be due to trauma or carbon dioxide during laparoscopic surgery which raises abdominal pressure, usually without a history in the neonatal period [8]. Further, ABH is said to have a deep relationship with body mass index [9]. In this case, the patient did not have a history of trauma or abdominal surgery. Obesity was considered to be one of the causes of hernia. Unlike infants who lapse into severe dyspnea soon after birth, the most frequent symptoms in ABH patients are mild discomfort such as chest tightness, abdominal discomfort, and dyspnea on effort [10]. Twenty-five percent of ABH patients do not complain about any symptoms [10]. Sagittal and coronal scan of enhanced CT with contrast media is useful for diagnosis. This provides detailed information about herniated viscera and diaphragm defects. In addition, the chest CT reveals the filled intestinal segments or the presence of soft tissues on the diaphragm and helps in making a definitive diagnosis of ABH. MRI was also reported as useful for depicting hernia and diaphragm defects [11].

For surgical treatment of Bochdalek hernias, both transabdominal and transthoracic approaches have been reported [2, 4, 12, 13]. If the patient had signs of intestinal obstruction or strangulation, an abdominal approach might be preferable to reintroduce the intestinal tract, resect ischemic organs, and reconstruct [2]. Meanwhile, if the protruded organs are suspected fatty tumors, a transthoracic approach might be an easier procedure for separating adhesions, resecting tumor, and repairing the diaphragm, especially if it is right-sided. Minimal invasive approaches by complete thoracoscopic surgery were also reported [14]. Proper surgical procedures should be selected due to the result of preoperative image examination.

In many cases, the hernia sac is returned to the abdomen to avoid pleural injury by incising the hernia sac [15]. Although the risk of seroma was reported in the remnant sac, it had been reported that the remnant sac disappeared after surgery [16]. Moreover, several reports said that surgeons tried to reduce the remnant sacs [15, 16]. In our case, there was no hernia sac because it was just a sliding hernia of the retroperitoneal fat pad through the Bochdalek foramen into the thoracic cavity. Many surgeons prefer to construct a repair that is reinforced with a prosthetic graft because of the continuing stress on the diaphragm that results from respiratory movements [17]. However, if the diaphragm defect is not so large, it may be better to construct the diaphragm by direct suturing to avoid infection and postoperative adhesions.

## Conclusions

ABH is a relatively rare disease, and the diagnosis is often difficult because of its poor symptoms. Careful examination is essential to determine the best surgical procedure. A transthoracic approach is useful if the ABH is right-sided.

## Abbreviations

ABH: Adult Bochdalek hernia
CT: Computed tomography
MRI: Magnetic resonance imaging
